# A Review of Current Techniques in Lip Reposition Surgery for Treating Excessive Gingival Display

**DOI:** 10.7759/cureus.75293

**Published:** 2024-12-07

**Authors:** Turki M Abu Alfaraj, Renad E Aljohani, Fayafi A AlFaifi, Orjwan S Mattar, Thekra Y Algasim, Raghad M Alghamdi, Jarman A Alasmari, Abdulaziz A Alzahrani

**Affiliations:** 1 Periodontics, Dental Department, Ministry of National Guard Health Affairs, Al-Madinah al-Munawwarah, SAU; 2 Dentistry, Ministry of Health, Al-Madinah al-Munawwarah, SAU; 3 General Dentistry, Jazan University, Jazan, SAU; 4 Dentistry, Ibn Sina National College for Medical Studies, Jeddah, SAU; 5 Dentistry, King Khalid University, Abha, SAU; 6 General Dentistry, College of Dentistry, King Saud bin Abdulaziz University for Health Sciences, Riyadh, SAU; 7 General Dentistry, King Abdullah International Medical Research Center, Ministry of National Guard Health Affairs, Riyadh, SAU

**Keywords:** botulinum toxin in lrs, excessive gingival display, hyperactive upper lip, lip reposition surgery, vertical maxillary excess

## Abstract

Excessive gingival display (EGD), commonly known as a gummy smile (GS), is a cosmetic concern that involves exposing a significant area of gum tissue during a smile, rendering it unaesthetic. Gingival exposure greater than 3 mm is deemed aesthetically displeasing and often necessitates treatment to mask the gummy smile. The causes of EGD are multifactorial, including altered passive eruption (APE), hypermobile upper lip (HUL), short lip length, increased vertical maxillary component, gingival hyperplasia, dentoalveolar extrusion, and more. As each aetiology requires different treatment modalities, patients with EGD should be thoroughly evaluated and individualized treatment plans should be developed. Lip repositioning surgery (LRS) is a minimally invasive conservative technique that decreases gummy smiles in patients with increased lip mobility and mild vertical maxillary excess. If the aetiology is multifactorial, LRS can be combined with other treatment modalities such as crown lengthening and gingivectomy for improved aesthetic outcomes. This review discusses the recent modifications and current techniques that have evolved from traditional LRS.

## Introduction and background

Smiling is an innate behavior in humans [1-3]. Not only does a smile enhance an individual’s beauty, self-confidence, and motivation, it also plays a crucial role in social interactions [1-3]. However, excessive gingival display or gummy smile (EGD/GS) can negatively impact a smile by making it visually unappealing [1-3]. Generally, a small amount of gingival exposure is acceptable for an aesthetic smile. A few authors also report that 1-3 mm of gingival visibility is regarded as the “ideal smile” [1-3]. Individuals with a high smile line that results in gingival visibility exceeding 2-4 mm while smiling are classified as EGD/GS patients [4-7]. While the specific amount of gingival visibility deemed unaesthetic in a smile varies across different populations, a gingival display exceeding 3 mm is regarded as unattractive globally [8-10]. The maxillary frontal area, lips, gums, and teeth are the anatomical landmarks that preserve a symmetric cosmetic harmony during a smile [4,11]. The etiologies of EGD/GS are described in Table 1 [2,4,11-13].

**Table 1:** The etiologies of gummy smile Information collected from sources [2,4,11-13].

Developmental/Skeletal	Anatomic/Soft Tissue	Dental	Pathologic
1. Vertical maxillary excess (VME)	1. Short upper lip	1. Dentoalveolar extrusion	1. Drug-induced gingival hyperplasia
2. Altered passive eruption (APE)	2. Upper lip asymmetry	2. Short clinical crown	
	3. Hyperactive upper lip (HUL) mobility		

A recent study reported that EGD/GS affects 7% of the adult population [14] and is more prevalent among females [14,15] and individuals of Black ethnic origin [14]. Given its multifaceted nature, the therapeutic strategy of choice for GS/EGD is determined by its underlying aetiology. Among the various aetiologies previously mentioned, hypermobile upper lip (HUL) and altered passive eruption (APE) are the two predominant causes of EGD/GS. Among adults in North America [16] and Asia [15] presenting with EGD/GS, over 85% exhibit HUL. Approximately 40% of cases are caused by HUL of isolated soft tissue origin, while 35-40% of cases are due to a combination of HUL and APE [15,16]. The presence of reduced upper lip length [14,15] and asymmetric upper lips [16,17] was noted to be rare (occurring in less than 10%) among individuals diagnosed with EGD/GS.

Lip reposition surgery (LRS) was first mentioned in plastic surgery literature by Rubinstein and Kostianovsky in 1973 [18]. It was later introduced in dentistry following its modification in 2006 by Rosenblatt and Simon [19]. LRS was recommended in particular instances of EGD/GS linked to HUL, shortened upper lip, and cases of mild vertical maxillary excess (VME) [11,20,21]. Orthognathic surgery is the primary treatment for vertical maxillary excess [22]; however, due to its complexity, invasiveness, and postoperative morbidity, patients tend to avoid this procedure [23,24]. This is where the less invasive LRS becomes relevant in mild VME cases.

LRS is a permanent non-invasive technique to address EGD/GS. This surgery restricts the muscle pull (zygomaticus minor, levator anguli, orbicularis oris, and levator labii superioris) responsible for smiling by decreasing the depth of the maxillary vestibule [9]. The LRS outlined in the plastic surgery literature in 1973 spares muscle manipulation and entails the removal of a band of mucosa from maxillary vestibule [18], thereby impeding the retraction of the upper lip elevator muscles. More invasive modifications and additions have since been implemented to ensure predictable stability and prevent relapse. These include separating the muscle attachment from the underlying structure [22], rhinoplasty-assisted lip elongation [25], incorporating a silicone spacer [26], myotomy of the levator labii superioris with frenectomy [27], and subperiosteal dissection of the gingiva [28]. LRS is not advised when VME exceeds 8 mm or when there is a reduced width of the attached gingiva, as this negatively impacts the design, stabilisation, and suturing of the flap [15,29].

In recent years, advancements in lip reposition surgery techniques have expanded the options available for managing excessive gingival display caused by hyperactive upper lip, vertical maxillary excess, and other anatomical or developmental factors. From modifications in incision placement to the integration of botulinum toxin and laser technologies, each approach offers unique benefits and addresses specific etiologies of the gummy smile. This review aims to comprehensively examine the current techniques, clinical outcomes, and emerging innovations in lip reposition surgery techniques, providing insights into the most effective strategies for achieving optimal aesthetic results.

## Review

Modifications to the traditional LRS technique and its historical evolution

One of the primary causes of EGD/GS is HUL. HUL refers to cases where the upper lip mobility surpasses 8 mm as measured from the resting position to the maximum extent of a smile [2,11]. It is commonly treated surgically using the LRS approach [30].

Early development of LRS techniques

The first LRS described in 1973 involves the excision of a split-thickness mucosal flap from the labial alveolar mucosa. This is followed by approximating the coronal (2-3 mm coronal to the mucogingival junction (MGJ)) and apical (at the depth of the vestibule) wound borders via suturing, resulting in a shortened vestibule. This method performs labial frenulum reconstruction by incorporating a fold in the reflected flap. The initial literature on LRS presented this surgical approach as a less invasive option compared to orthognathic surgery [18].

In 1979, Litton and Fournier [22] revisited the original LRS technique and implemented a modification that involved elevating a full-thickness flap instead of a partial-thickness flap, while also avoiding frenulum reconstruction. They emphasized muscle detachment in cases where the lip exhibited a shorter length. Later, the LRS referenced in the dental literature in 2006 [19] and 2007 [29] adhered to the traditional technique [18], which involves removing a strip of partial thickness mucosal flap without frenulum reconstruction. The coronal incision height is at the level of the MGJ, while the apical incision is at the level of the vestibule depth. The coronal and apical incisions run parallel to each other and connect on both sides at the level of the commissure of the lip when smiling. The position of the lip commissure projection can differ between the second premolar and the first molar, influenced by the width of the patient's smile [30].

Modifications in the position of coronal and apical incisions

Several modifications have been proposed for the positions of the two horizontal parallel incisions (coronal and apical) in LRS. These modifications are summarized in Table 2 [10-45].

**Table 2:** Summary of Coronal and Apical Incision Modifications in Lip Reposition Surgery by Various Authors MGJ = Mucogingival Junction

Authors	Modifications
Suh JJ et al. [31], Chacon G [32]	Coronal incision positioned 1-2 mm apical to the mucogingival junction (MGJ), with the apical incision located 10-12 mm from the coronal incision or at the transition line between the masticatory mucosa and labial line.
Rosenblatt A et al. [19], Simon Z et al. [29], Bhola M et al. [20], Ozturan S et al. [33], Tawfik OK et al. [34], Jacobs and Jacobs [36], Torabi A et al. [37], Zardawi F et al. [38], Duruel O et al. [39], Vergara-Buenaventura A et al. [40]	Coronal incision placed at the level of MGJ.
Silva C et al. [41], Alammar A et al. [42], Ribeiro-Júnior NV et al. [10], Littuma G et al. [43]	Coronal incision placed 1 mm coronal to MGJ.
Kamer F et al. [44], Litton C et al. [22], Rubinstein and Kostianovsky [18]	Coronal incision placed 2-3 mm coronal to MGJ or 3-4 mm apical to the gingival margin, with the apical incision made at the fundus of the upper buccal sulcus.
Zardawi F et al. [38], Aly L et al. [35]	Coronal incision made at the MGJ, extending either 5 mm or 6-8 mm upward into the vestibule, with the apical incision positioned 5 mm from the coronal incision.
Littuma G et al. [43], Abdullah WA et al. [45]	Coronal incision placed 4-5 mm apical to the gingival margins, extending from one second premolar to the other, with the apical incision 8-10 mm further apical on the labial mucosa.
Bhola M et al. [20], Tawfik OK et al. [34], Simon Z et al. [29], Humayun et al. [46], Jacobs and Jacobs [36], Torabi A et al. [37], Duruel O et al. [39], Vergara-Buenaventura A et al. [40]	Apical incision placed at double the amount of gingival display (in mm) during a smile from the coronal incision.
Ribeiro-Júnior NV et al. [10], Alammar A et al. [42], Rosenblatt A et al. [19], Simon Z et al. [29]	Apical incision placed not greater than 10-12 mm from the coronal incision.

In addition to considering the apico-coronal incision dimensions, the surgeon must also account for the reduction of gingival visibility during a smile, the flap design that preserves 2-3 mm of keratinized attached gingiva, and the maintenance of vestibular depth to ensure proper masticatory and lip functions. Furthermore, avoiding involving the vermilion border and related anatomical landmarks is crucial during flap design [30]. LRS results in an increased upper lip length or fullness while smiling [30,41,47], which promotes a socially pleasant smile with a compelling aesthetic factor [48], and should not change the lip dimensions at rest [47].

Recent modifications in LRS

In LRS, the amount of mucosal band removal is typically determined by the gingival visibility in the central incisor area as a reference point. In 2020, Duruel et al. [39] conducted targeted tooth-based LRS surgery, where gingival visibility is assessed individually for each tooth, and the amount of mucosa removed is twice the individual tooth's gingival visibility (in mm) during a smile. This case report used a short-term follow-up of three months, with gingival visibility measuring less than 3 mm post-operatively. Further long-term trials with larger populations are necessary to investigate the benefits of this technique more thoroughly [39].

The use of lasers instead of stainless-steel blades in LRS surgery is referred to as laser-assisted LRS. The benefits of employing modern technological tools, such as diode and erbium lasers, as surgical instruments include minimal invasiveness, bloodless operative fields, enhanced surgical visibility, and reduced post-surgery pain and inflammation. Consequently, this leads to a decreased need for analgesics and lowers the risk of relapse compared to conventional techniques [49]. The laser was first used as an adjunct to LRS in 2013 to mark the outline of the incision, intending to temporarily insert a suture needle with thread to simulate a mock LRS procedure. This helps the patient anticipate the exact position of the lip and the extent of gingival display reduction during their smile before the actual surgery [36]. Some clinicians have utilised diode lasers as an outline tool before proceeding with the surgery [50]. Diode lasers [33,51] and erbium lasers [31] are used in LRS to demarcate the outline of incisions and excise the band of mucosa within the outlined area. A few authors also used diode and erbium lasers for outlining incisions and ablating the outlined band of mucosa [49,52-54]. There are several case reports on laser use, and yet there is no evidence-based support from long-term studies with large populations to demonstrate that laser LRS is superior to conventional LRS.

Botulinum toxin injections with LRS

The use of botulinum toxin (BTX) injections as an adjunct to LRS is another modification mentioned in the literature. The main purpose of administering BTX with LRS is to reduce muscle tension during the post-operative healing phase and therefore avoid scarring [55]. BTX was initially used for surgeries involving upper lip anatomic landmarks like cheloplasties [56] and cleft lip surgery [57]. The application of BTX in these surgical interventions yielded minimal scarring and superior scar quality [57]. Aly and Hamouda first introduced BTX into LRS by applying BTX two weeks post-LRS [35]. Other authors have followed different timelines for BTX application, such as one day after LRS [40], two weeks before LRS [58], three months before LRS, and two weeks post-operatively after LRS [59]. Recently, one author employed BTX two weeks before LRS, followed by repeated BTX injections at specific intervals (two, four, and eight months) after LRS [60]. The maximal impact of BTX injection is observed at 48 hours, with the resultant effects persisting for a duration of three to six months [61,62]. Therefore, when employing LRS to alleviate muscle tension during the initial postoperative healing phase, it is advisable to administer BTX injections at least 48 hours prior to LRS and not exceed a few weeks [55]. In 2022, Antunes et al. [58] performed a controlled trial and advised application of BTX 15 days before LRS for better post-surgical outcomes [58]. Furthermore, BTX can be applied as an adjunct to LRS in cases with moderate VME and HUL, where surgical intervention alone would likely produce insufficient results and suboptimal aesthetic outcomes following the procedure [35]. Repeated BTX injections after LRS will also relax the upper lip elevator muscles for an extended period, encouraging stable outcomes without relapse. This approach provides long-term results and also avoids invasive procedures, like myotomies, to manage relapse [60].

Surgical considerations in muscle management and suturing techniques

There are several modifications related to surgical considerations. These range from full-thickness to partial-thickness flaps that use either blunt or sharp dissection methods, various muscle management techniques, and innovative suturing methods.

*Early Muscle Detachment Techniques and Myotomy* In the late 1970s, Litton and Fournier [22] performed full-thickness flap elevation in LRS. As previously mentioned, they recommended muscle detachment for cases involving a shorter upper lip [22]. The original LRS technique led to relapses; therefore, Miskinyar [63] modified the technique by incorporating the amputation of the levator labi superioris muscle [63]. Since muscle dissection reduced EGD/GS, other authors subsequently incorporated the full-thickness flap and myotomy in LRS. These additions alter the position of the smile muscle, restrict muscle pull, and ultimately lead to better results with less relapse, which will be discussed in the forthcoming sections.

Advanced Suturing Techniques and Polyester Thread Use

Full-thickness flap elevation with blunt dissection of muscle and the use of bilateral suspensory sutures was proposed in 2014. Sutures were secured around the canines, and the released levator labii superioris and depressor septi muscles were relocated to a more inferior position [45]. A similar surgical method with periosteal fenestration and suspensory sutures through attached gingiva was performed later [37]. A comparative interventional study incorporating the similar techniques discussed above versus conventional LRS showed minimal relapse with the modified method [42]. In another study, the elevation of split-thickness flaps, followed by muscle blunt dissection and the use of continuous periosteal sutures for the first time to prevent relapse, demonstrated improved stability of the outcomes [34]. The synergistic effects of full and partial-thickness flaps, combined with blunt muscle dissection and advanced periosteal suturing, have been reported [32]. Post-operative dehiscence and more frequent, prolonged episodes of short-term numbness are clear consequences of full-thickness flap elevation [42].

Myotomy and Frenectomy Techniques

Ishida et al. [27] performed a myotomy of the levator labii superioris through subperiosteal dissection via lateral incisions in the columella of the nostril, which diminished the action of the upper lip elevator muscle. Along with this, they performed a frenectomy that resulted in lengthening of the upper lip [27].

Muscle Containment and Suturing Techniques

Muscle containment through the suturing technique is a modification that is incorporated in both conventional [29] and modified LRS [41]. In a comparative study, internal periosteal sutures were performed on identical twin subjects during LRS. At the three-year follow-up appointment, both groups-with and without internal periosteal sutures-demonstrated better outcomes. However, the periosteal suture group exhibited greater outcome stability and patient satisfaction [64]. The internal horizontal mattress sutures performed in the LRS case series demonstrated improved stability and a greater ability to restrict muscle pull; these benefits were evident at seven years post-operatively in one of the cases [50]. In another comparative study, muscle traction was performed using internal dual suturing in modified LRS. Comparing this group with modified LRS alone demonstrated a satisfactory aesthetic outcome and stability at the one-year follow-up [65]. In a recent comparative study, dual-layer suturing with modified LRS was compared to modified LRS alone. The group receiving dual-layer suturing showed no long-term benefits; however, a complete relapse in surgery was delayed by up to six months, while the modified LRS alone group experienced a complete relapse at three months [66]. Since these procedures are less invasive compared to techniques that involve myotomy and full-thickness flap elevation, long-term studies with large populations are necessary to explore the potential benefits of both these procedures. In a recently reported case study, modified LRS with an internal horizontal mattress suture and simple external suture followed by aesthetic crown lengthening was performed. It resulted in a partial relapse of baseline gingival visibility at six-year follow-up [67]. Securing the lip stably in its newly relocated position is the primary factor affecting the success of LRS. Hence, it is recommended to use the proper suturing technique for an adequate period to restrict the pull of upper lip elevator muscles [30,66,68,69].

Another modification is the use of polyester threads as an adjunct to LRS. Polyester threads have been used to minimise muscle activity and relapse post-operatively after LRS [70,71]. These threads, which are inserted through injections, act as physical blockades that hinder muscle mobility and reattachment. One thread is positioned horizontally on the bone in the sub-nasal region, while the remaining two threads are placed bilaterally near the canine fossa [70,71]. One month post-operatively after LRS (either through muscle detachment or muscle containment via suturing), these polyester threads are inserted with BTX injection 15 days before LRS [71] or without BTX [70]. There is no concrete evidence that inserting these threads or adding multiple threads yields the expected outcome of minimising muscle activity and relapse after LRS.

Modifications for Maxillary Lip Asymmetry

Modifications made in LRS for maxillary lip asymmetry cases involve adjusting the size of the mucosal strip removed on each side, depending on the extent of gingival visibility. Occasionally, the mucosal band is removed unilaterally, based on the specific needs of the patient [20,37]. If the aetiology of EGD/GS is multifactorial, the implementation of LRS alongside supplementary procedures such as cosmetic crown lengthening and gingivectomy is warranted. However, it requires careful treatment planning and proper sequencing of interventions to achieve improved aesthetic outcomes [32,72,73].

Frenum-Sparing Techniques

Another modification during LRS is to spare the frenum (a thick band of midline mucosa) by excising only the bilateral strips of mucosa without encroaching on the midline frenum [10,20,37,41,74]. This modification reduces postoperative morbidity and maintains the midline. EGD/GS reduction is more effective in cases treated with LRS without frenectomy [13]. However, in certain situations where the frenulum is close to the gingival margin, it can exacerbate the gummy smile due to increased upward pull of the maxillary lip. In such cases, LRS with frenectomy is recommended for improved outcomes by repositioning the frenum to a more favourable location [75,76].

Recent Modifications: Double M-V Plasty and Filler Use

The most recent modification in the literature reported double M-V plasty LRS. Midline double V-plasty facilitates the anatomical relocation of the frenulum into a favourable position, while bilateral vertical M-plasty permits more extensive mucosal excision and improved approximation of the wound margins without soft tissue defects along the incision lines. Compared to the conventional approach, it results in a more aesthetic and functionally viable LRS [77].

The use of polymethylmethacrylate-based cement spacers or implants [78-80] and fillers such as lipoaspirate [81] and hyaluronic acid [82] minimises muscle movement during a smile and helps reposition the lip to reduce EGD/GS. However, they are not used as part of LRS.

Table 3 shows descriptions and outcomes of various modifications in lip reposition surgery.

**Table 3:** Overview of Modifications in Lip Reposition Surgery Techniques and Their Outcomes LRS: Lip Reposition Surgery MGJ: Mucogingival Junction HUL: Hyperactive Upper Lip BTX: Botulinum Toxin PMMA: Polymethylmethacrylate EGD/GS: Excessive Gingival Display / Gummy Smile

Modification	Description	Benefits/Outcomes
Original LRS [18].	Excision of a split-thickness mucosal flap with frenulum reconstruction; aimed to shorten the vestibule.	Less invasive than orthognathic surgery; aimed to address HUL.
Litton and Fournier modification [22]	Elevated full-thickness flap and emphasized muscle detachment for shorter lip cases; avoided frenulum reconstruction.	Avoided frenulum issues, accommodated short lip cases; enhanced stability by reducing muscle pull.
Traditional LRS without frenulum reconstruction [19,29]	Used a partial thickness mucosal flap removal without frenulum reconstruction; aimed at MGJ and vestibule depth.	Adheres to initial LRS goals, retains simplicity without frenulum involvement.
Targeted tooth-based LRS [39].	Assessed gingival visibility for each tooth, removing twice the visibility in mm for each tooth.	Reduced gingival visibility under short follow-up; needs larger studies.
Laser-assisted LRS [31,33,36,49-54].	Utilizes diode and erbium lasers to outline and excise mucosa; offers minimal invasiveness and reduced post-op pain.	Minimally invasive, bloodless, and with reduced relapse risk; patient can preview post-op smile.
Botulinum toxin (BTX) injections with LRS [55,60]	BTX used to relax muscles, reduce scarring; applied at various intervals pre- and post-LRS to enhance stability.	Enhanced muscle relaxation during healing, less scarring, better stability, and minimized relapse.
Full-thickness flap elevation with blunt dissection and suspensory sutures [45].	Proposed muscle relocation and periosteal fenestration with sutures around canines to improve outcomes and reduce relapse.	Enhanced muscle control and lip positioning, minimal relapse, stable post-op outcomes.
Comparative interventional study on periosteal sutures [64].	Compared outcomes of periosteal sutures versus traditional LRS, showing improved stability and minimal relapse.	Improved stability and outcome satisfaction compared to traditional LRS.
Delay in relapse with double-layered suturing [66].	Incorporated continuous periosteal sutures to prevent relapse, with improved outcome stability.	Better long-term outcome stability, minimized relapse.
Use of polyester threads to minimize muscle activity post-LRS [70,71].	Polyester threads injected post-LRS to act as physical barriers, reducing muscle reattachment and relapse.	Inhibits muscle reattachment, longer-lasting outcome stability post-LRS.
LRS with frenectomy [75,76] .	Frenectomy combined with LRS to reposition frenum, beneficial for cases where frenum causes gummy smile.	Improves results in cases with prominent frenulum; reduces muscle pull-induced gummy smile.
Double M-V plasty LRS [77].	Midline double V-plasty and bilateral M-plasty for more extensive mucosal excision, preserving soft tissue aesthetics.	Preserves soft tissue, enhances aesthetic outcome without soft tissue defects.
Use of PMMA-based cement spacers or implants, lipoaspirate, and hyaluronic acid fillers [78–82] .	Not part of LRS but used to minimize muscle movement and adjust lip positioning in EGD/GS management.	Temporary measure; adds aesthetic control but not a surgical solution.

Discussion

As previously mentioned, among the various therapeutic approaches for EGD/GS, LRS remains the primary treatment option. This is mainly because the most prevalent aetiology for EGD/GS is HUL, which is effectively managed through LRS. In an initial systematic review published in 2018, LRS was found to reduce EGD/GS by an average of 3.4 mm [9]. Another systematic review showed an EGD/GS reduction of 2.87 mm after three months of LRS, which decreased to 2.71 mm after six months and 2.10 mm after 12 months [83]. In their systematic review and meta-analysis, Younespour et al. [13] revealed that after LRS with labial frenectomy, full-thickness flap, and myotomy, the average amount of gingival display decreased significantly from the baseline measurements taken before the procedure. Specifically, the mean reduction was 2.98 mm at three months post-treatment and 2.90 mm at six months. In patients who underwent LRS with frenectomy, partial-thickness flap, and without myotomy, the EGD/GS reduction was 2.68 mm at six months and 2.52 mm at 12 months. In another modality where the patients received LRS without frenectomy, partial-thickness flap, and without myotomy, the EGD/GS reduction was about 3.22 at six months. The surgical protocol of LRS without frenectomy showed better gingival visibility reduction [13]. In a recent systematic review and meta-analysis, four out of 11 included studies reported EGD/GS reduction that lasted up to the postoperative 12-month follow-up. Meta-analysis revealed that the mean decrease in EGD/GS at one, three, six, and 12 months was 3.64 mm, 3.34 mm, 2.94 mm, and 2.29 mm [84].

In their controlled trial study, Tawfik et al. [34] reported an EGD/GS reduction of 2.73 mm with traditional LRS and a 3.57 mm reduction with gingival visibility in LRS with myotomy. Enhanced outcomes and greater patient satisfaction were observed with the combination of LRS and myotomy compared to LRS alone [34], and this finding aligns with another comparative study [42]. It is evident from the above studies that the improvement in EGD/GS reduction decreases over a while. LRS incorporated with myotomy and LRS without frenectomy showed a greater decrease in EGD/GS. According to several studies, LRS in conjunction with myotomy leads to better clinical outcomes with minimal relapse [21,85-87]. Despite the limited literature, there are positive inferences from various studies supporting myotomy. This necessitates further long-term, large-population comparative clinical trials to assess the efficacy of myotomy in comparison to conventional techniques [87].

A randomised controlled trial involving a large population (n=200) compared the clinical outcomes and long-term stability of conventional LRS and modified LRS with periosteal suturing. At the one-year follow-up, the modified LRS approach showed improved stability with no reported relapses. Gingival visibility one year after LRS was 3.77 mm (conventional LRS) and 2.48 mm (modified LRS with periosteal suturing). The study concluded that a 2-3 mm reduction in EGD/GS is attainable with modified LRS [69]. Additionally, improved patient satisfaction was reported with the results of modified LRS compared to conventional LRS [88].

## Conclusions

The studies discussed in the current review have found that modified LRS leads to increased patient satisfaction. The overall reduction in gingival visibility with modified LRS is reported to be 2-3 mm. As gingival visibility increases postoperatively at monthly intervals, the EGD/GS reduction after LRS is decreased, which can lead to a relapse. Numerous modifications, such as myotomy, muscle containment through sutures, and the adjunctive use of BTX and polyester threads, can help mitigate or delay these relapses. Future research should focus on identifying more minimally invasive modifications of LRS to reduce complications associated with surgery, including advancements in suturing techniques, optimal timing for the adjunctive use of botulinum toxin, and the use of lasers. Since most articles in the existing literature on GS/EGD reduction or LRS are case reports and case series, further long-term, large-population comparative studies and clinical trials are essential to explore clinical and patient-related outcomes, as well as the long-term stability and viability of the emerging LRS modifications. Additionally, exploring alternative treatment approaches, such as spacers and fillers, could further enhance treatment efficacy in EGD/GS.
